# Circadian clock associates with tumor microenvironment in thoracic cancers

**DOI:** 10.18632/aging.102450

**Published:** 2019-12-23

**Authors:** Yong Yang, Guangda Yuan, Hongya Xie, Tengteng Wei, Donglin Zhu, Jianyong Cui, Xiaoqiang Liu, Rongming Shen, Yimeng Zhu, Xuefang Yang

**Affiliations:** 1 Department of Thoracic Surgery, The Affiliated Suzhou Hospital of Nanjing Medical University, Nanjing Medical University, Jiangsu 215001, China; 2 The Affiliated Suzhou Hospital of Nanjing Medical University, Nanjing Medical University, Jiangsu 215001, China

**Keywords:** circadian clock, tumor microenvironment, immune cells, multi-omics, immunotherapy

## Abstract

The application of cancer chronotherapy is to treat cancers based on at specific times during circadian rhythms. Previous studies have characterized the impact of circadian clock on tumorigenesis and specific immune cells. Here, by using multi-omics computation techniques, we systematically characterized the distinct roles of core circadian clock genes in thoracic cancers including lung adenocarcinoma, lung squamous cell carcinoma, and esophageal carcinoma. Strikingly, a wide range of core clock genes are epigenetically altered in lung adenocarcinomas and lung squamous cell carcinomas but not esophageal carcinomas. Further cancer hallmark analysis reveals that several core clock genes highly correlate with apoptosis and cell cycle such as RORA and PER2. Interestingly, our results reveal that CD4 and CD8 T cells are correlated with core clock molecules especially in lung adenocarcinomas and lung squamous cell carcinomas, indicating that chrono-immunotherapy may serve as a candidate option for future cancer management.

## INTRODUCTION

The application of cancer chronotherapy is to treat cancers based on at specific times during circadian rhythms. Optimising the time of drug may offer advantages over the original one in the improvement of drug efficacy and safety without increasing drug doses and changing drug types. To date, significant progress has been made to unravel the circadian features of several drugs. A recent study reported the time-dependent effects of sulfasalazine on cancer cell, and administering xCT inhibitors based on circadian rhythm will improve anti-tumor robustness [1]. Another study reveals that the core circadian clock gene BMAL1 inhibits tumorigenesis and increases paclitaxel sensitivity in tongue squamous cell carcinoma [2]. Numbers of RCTs and clinical practices also highlight the feasibility and validity of circadian-based treatments [3], because only the dosing time of the existing agents needs to be changed. However, the absence of a systematic computation for circadian timing in cancer therapies makes it a pressing challenge. Hence, it is required and urgent to further explore reliable circadian timing strategies.

In mammals, circadian clock is orchestrated through interlocked transcriptional-translational feedback loops. In the daytime, the Circadian Locomotor Output Cycles Kaput (CLOCK) and brain and Brain and Muscle Arnt-Like protein 1 (BMAL1, also named as ARNTL) were activated. Yet, period proteins (PER1, PER2, and PER3) and Cryptochrome protein (CRY1, and CRY2) shows upregulated expression at night, which thereafter repress the activity of CLOCK and BMAL1. Another loop down-regulates BMAL1, which is composed of Nuclear hormone Receptor subfamily 1 group D member ½ (NRD1/2, also named as REV-ERBa) and Retinoid-related Orphan Receptors (RORs). These feedback loops orchestrates the circadian rhythms in key life processes cell metabolism, inflammation and DNA damage response [4]. In the context of oncology, core circadian clock molecules were observed to modulate tumor progression and development [5–7]. Recently, Bu et al. reported a PERK-miR-211 axis which inhibits the circadian clock protein synthesis, and hence facilitating tumour progression [8]. Another study highlighted the lethal effects of the pharmacological activation of NRD1/2 [9]. Their results suggested that NRD1/2 could inhibit the autophagy and selectively exert antitumor effects against malignant and benign neoplasms [9]. Thus, those core circadian molecules largely represent the circadian rhythm state of samples.

It was long established that the immune system was tightly regulated by the circadian clock [4]. For example, Cao et al. reported that mice knocking out Cry1 and Cry2 unexpectedly displayed the autoimmune phenotype of higher serum IgG levels and antinuclear antibodies [10]. Interestingly, another independent group found that loss of BMAL1, which is another key component of circadian clock, induced T cell-associated CNS autoimmune diseases [11]. As for adaptive immune response, Druzd et al. demonstrated that responses to immunization and pathogens are time-dependent [12]. The number of lymphocytes in lymph nodes oscillates, where it peaked at night and further dropped in the daytime [12]. Most recently, the circadian clock was observed to block PD-L1 expression in activated macrophages and monocytes in sepsis [13]. Though it was based on the animal model of sepsis, this study underlies the potential application of immune-chronotherapy as for cancer treatments [13]. This interesting finding drives us to explore the interaction between immune checkpoints and circadian clock especially in cancer.

Thus far, how circadian clock shapes the tumor microenvironment and immune infiltrates in thoracic cancers (lung adenocarcinoma, lung squamous cell carcinoma, and esophageal carcinoma) still remains poorly defined. Recent progress in bioinformatics tools enabled transcriptome-wide studies of circadian clock at an unprecedented scale and resolution. Here, powered by multi-omics analysis, we aimed to answer the question how circadian clock core molecules regulates hallmark oncogenic pathways and the drug effectiveness. Through this approach, we demonstrate the crosstalk between tumor microenvironment and circadian clock, providing novel insights of the functional engagements of circadian clock in thoracic cancers.

## RESULTS

### Defining core circadian clock genes in thoracic cancers and normal tissue

**Figure 1:**
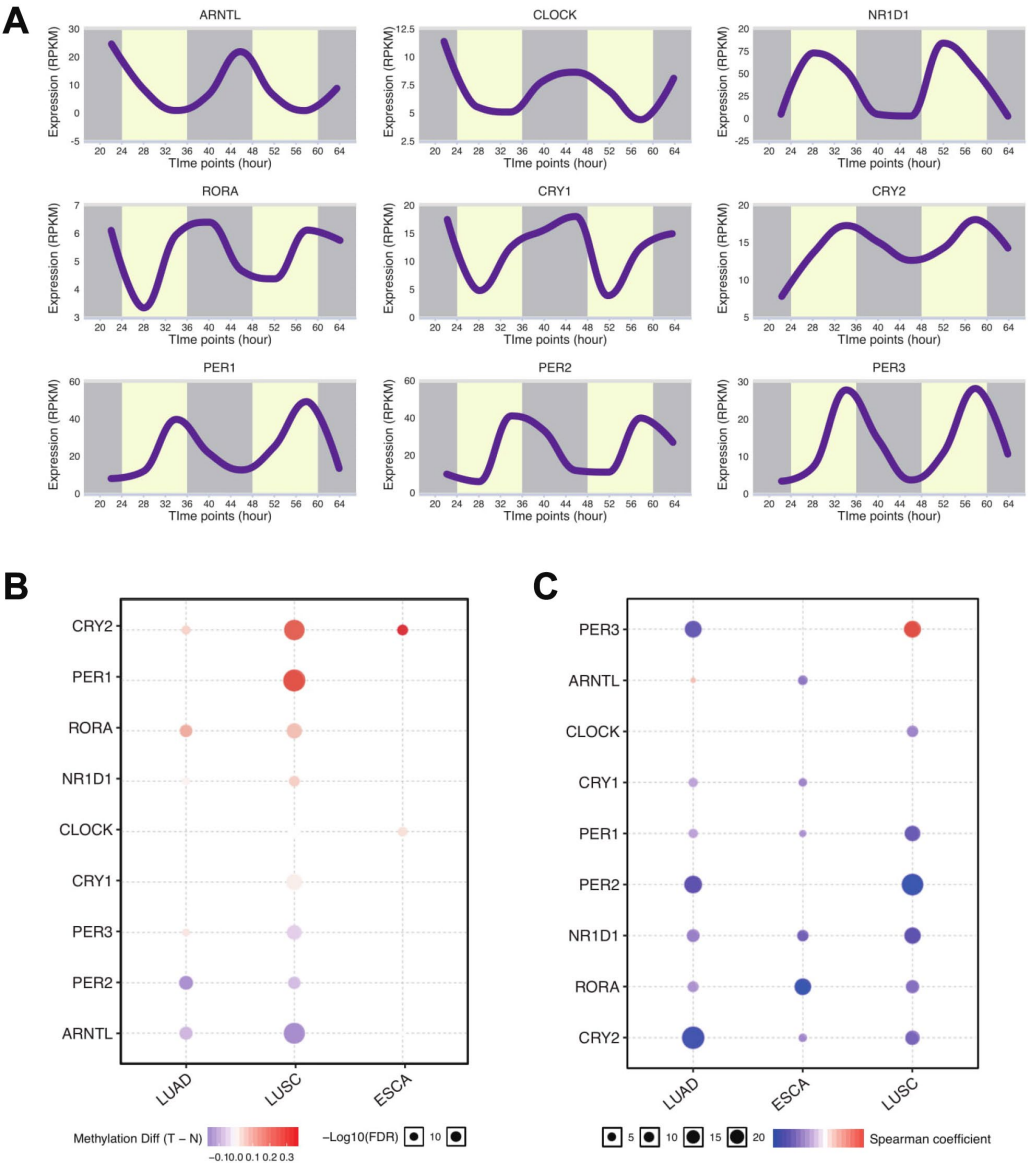
**Core circadian clock genes are altered in thoracic cancers.** (**A**) The circadian rhythm of core circadian genes, including CRY2, PER1, RORA, NR1D1, CLOCK, CRY1, PER3, PER2, and ARNTL. (**B**) The methylation difference between tumors and normal tissues. (**C**) The methylation level affects the core circadian gene expressions. Abbreviations: LUAD, lung adenocarcinoma; LUSC, lung squamous cell carcinoma; ESCA, esophageal carcinoma.

To explore the role of circadian clock in tumors, we first selected the core circadian clock genes, including CLOCK, BMAL1, CRY1, CRY2, NR1D1, PER1, PER2, PER3, and RORA based on the literature [14, 15], to characterize the circadian state of patients. In the normal tissue dataset (GTEX), PER1, NR1D1, and CRY2 were highly expressed in esophagus and lung tissues (Supplementary Figure 1). Next, we computed the expression profile of those core circadian genes of lung tissue at different timings by using RNA-seq [16] and observed the oscillation of those genes (Figure 1). We next retrieved data of lung adenocarcinoma (LUAD) patients, lung squamous cell carcinoma (LUSC) patients, and esophageal carcinoma (ESCA) patients from The Cancer Genome Atlas (https://cancergenome.nih.gov). We computed the methylation level between normal tissues and tumors. As for LUAD and LUSC, over a half of core circadian clock genes was differently methylated between tumors and controls (Figure 1). However, in ESCA, only CLOCK and CRY2 methylation levels were significantly different. We also found that methylation downregulated almost all the circadian clock gene expressions in thoracic cancers (Figure 1). These data supports that core circadian clock genes are epigenetically altered in thoracic cancers.

### Circadian clock associates with the cancer hallmarks in thoracic cancers

In order to evaluate the potential effects of disruption of circadian clock in thoracic cancer patients, we attempted to compute the activities of hallmark pathways including TSC/mTOR, RTK, RAS/MAPK, PI3K/AKT, Hormone ER, Hormone AR, EMT, DNA Damage Response, Cell Cycle, and Apoptosis pathways. We extracted data from The Cancer Proteome Atlas (http://tcpaportal.org/tcpa/) to compute activity scores for 10 cancer related pathways and 3 cancer types. RPPA data were normalized by standard deviation, and pathway score equals to the relative protein level of all positive-regulatory molecules minus negative-regulatory molecules (see Methods).

**Figure 2:**
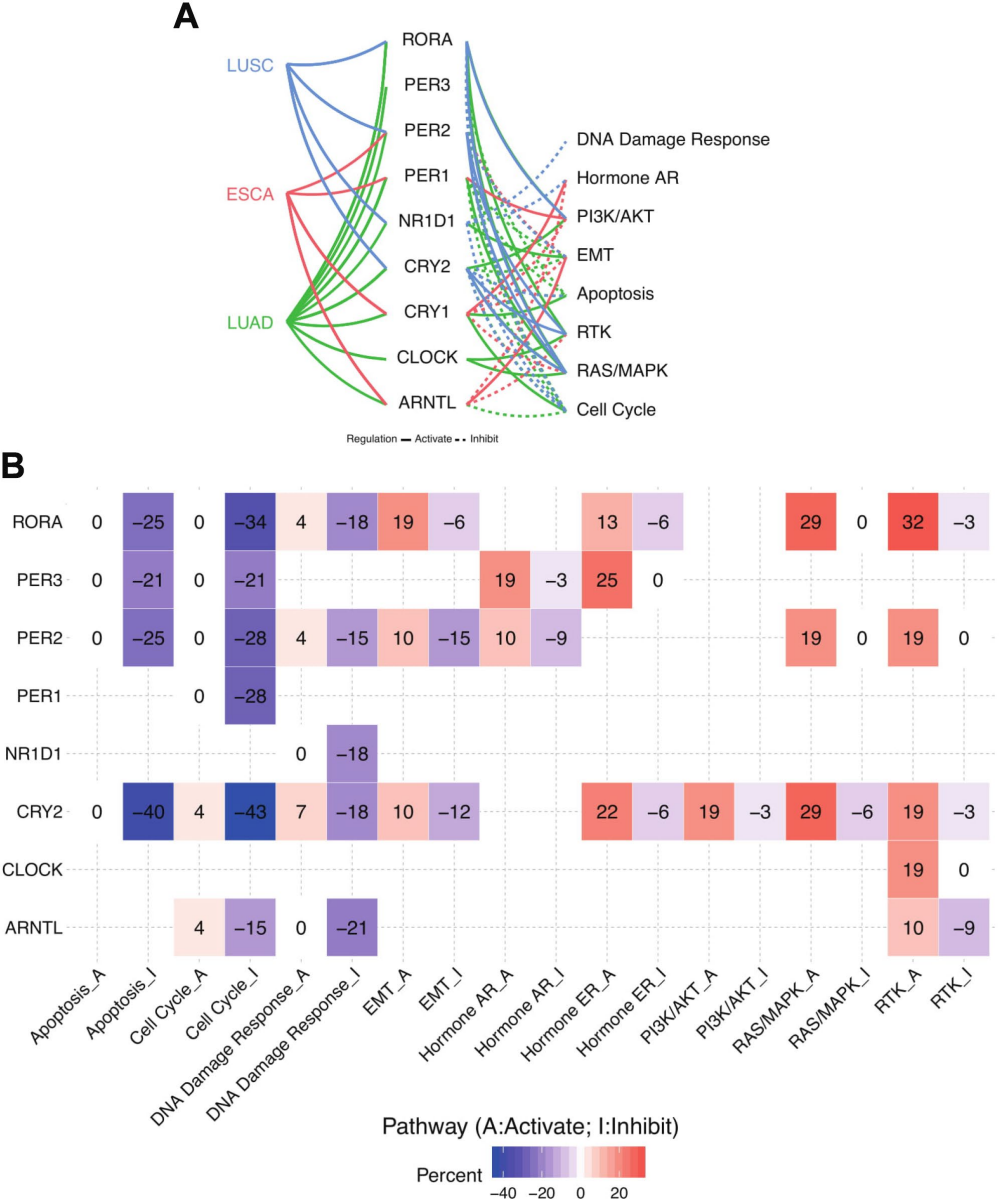
**Circadian clock widely impacts the cancer-related signalling pathways.** (**A**) Links between core circadian clock genes and hallmark signalling pathways. (**B**) The combined percentage of pathway activity. Abbreviations: LUAD, lung adenocarcinoma; LUSC, lung squamous cell carcinoma; ESCA, esophageal carcinoma); A, activate; I, inhibit.

As a result, circadian clock core genes widely associates with the hallmark cancer-related pathways especially in LUAD (Figure 2A). Generally, core circadian genes exert inhibitory effects on the cell cycle. In other words, disruption of circadian clock may trigger the dysfunction of cell cycle, thus inducing the tumor growth, which was in accordance with another independent group’s publication [17]. As for apoptosis pathway, the inhibitive function of circadian clock is predominant, where CRY2, PER2, and RORA play the leading role (Figure 2A). On the other side, our results also uncover unknown circadian clock-associated pathways. For example, we found a strong activation of RTK signalling pathways by core circadian genes in LUAD and LUSC. In ~32% of patients, RORA drives the upregulation of RTK related proteins. PER2, CRY2, and CLOCK also act as activators in this process. Hence, our observation not only confirmed previously reported circadian clock related hallmark pathways, but also discovered additional circadian clock regulated signalling pathways for thoracic cancers (Supplementary Figure 2).

### The clinical relevance of circadian clock genes

Previous evidence has shown that disruption of circadian clock could accelerate tumorigenesis and circadian genes were closely correlated with prognosis [5–7]. With a focus on thoracic cancers, we calculate the impact of circadian clock on patients prognosis (log-rank P value). Strikingly, we observed that several core circadian clock genes associate with the survival of patients. For example, higher expression of RORA and CRY2 associates with better survival in LUAD patients (Figure 2A, 2B). Similarly, PER1 shows similar trend in ESCA patients (Figure 2C). The full survival curve can be seen in Supplementary Figure 3. We also found core circadian clocks vary in LUAD and LUSC subtypes, and they could potentially serve as biomarkers for particular subtype prediction (Supplementary Figure 4).

**Figure 3:**
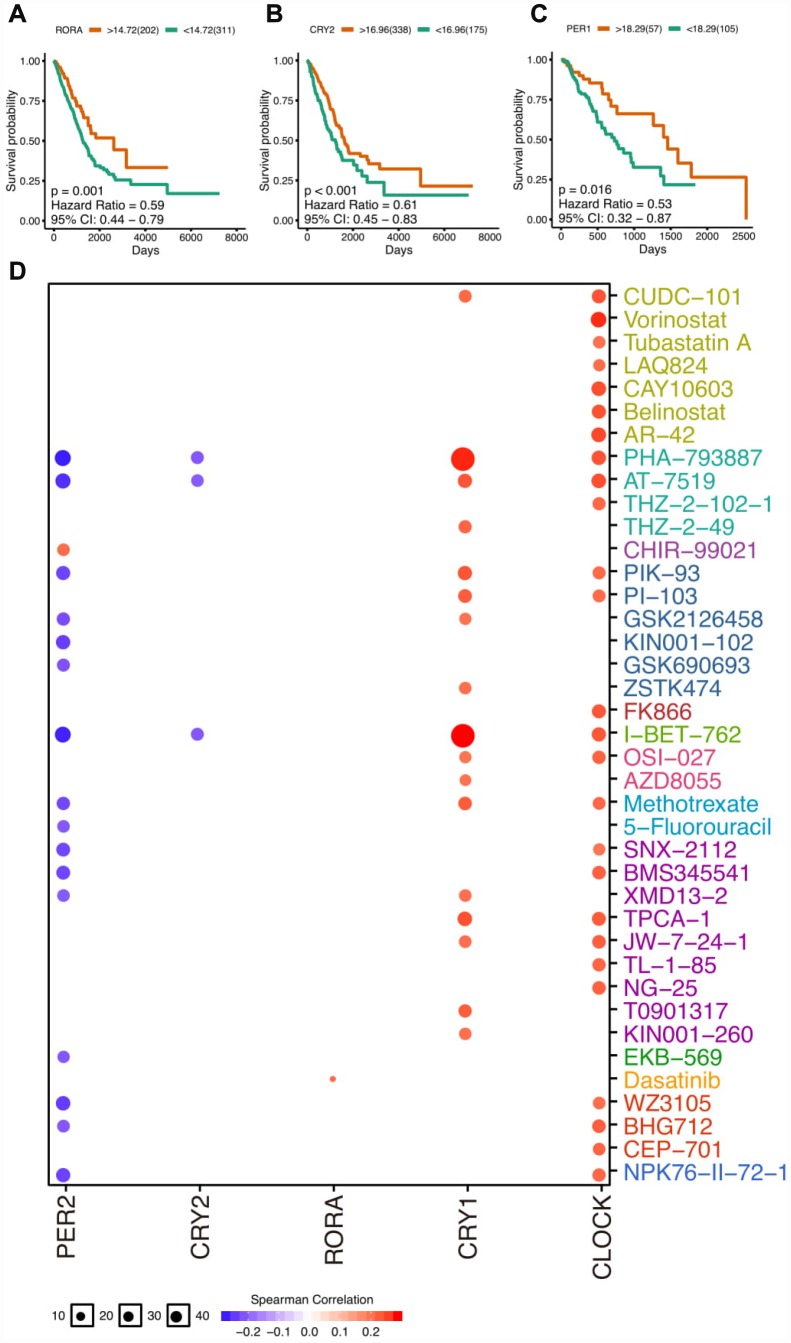
**The clinical relevance of core clock genes.** (**A**) The survival plot of RORA gene expression (log-transformed FPKM) in lung adenocarcinoma. (**B**) The survival plot of CRY2 gene expression (log-transformed FPKM) in lung adenocarcinoma. (**C**) The survival plot of PER1 gene expression (log-transformed FPKM) in esophageal carcinoma. (**D**) Correlation between drugs and core circadian clock genes. The full survival plots can be seen in supplementary materials.

To develop cancer chronotherapy, a crucial way is to assess the link between clock genes and existed drug targets. The clock-related drug administration following circadian timing may remarkably improve the effectiveness and reduce the toxicity. To analyze the correlation of expression and drug sensitivity, the Pearson correlation coefficients of transcript levels and AUCs was used and normalized based on Fisher’s Z transformation based on Cancer Therapeutics Response Portal (https://portals.broadinstitute.org/ctrp/) as was described before [16]. We observed that resistance (positive correlation) is associated with the expression of CLOCK and CRY1 (Figure 3D). On the contrary, PER2 were CRY2 were related with the drug sensitivity (negative correlation).

### Circadian clock correlates with the immune infiltrates in lung cancer

**Figure 4:**
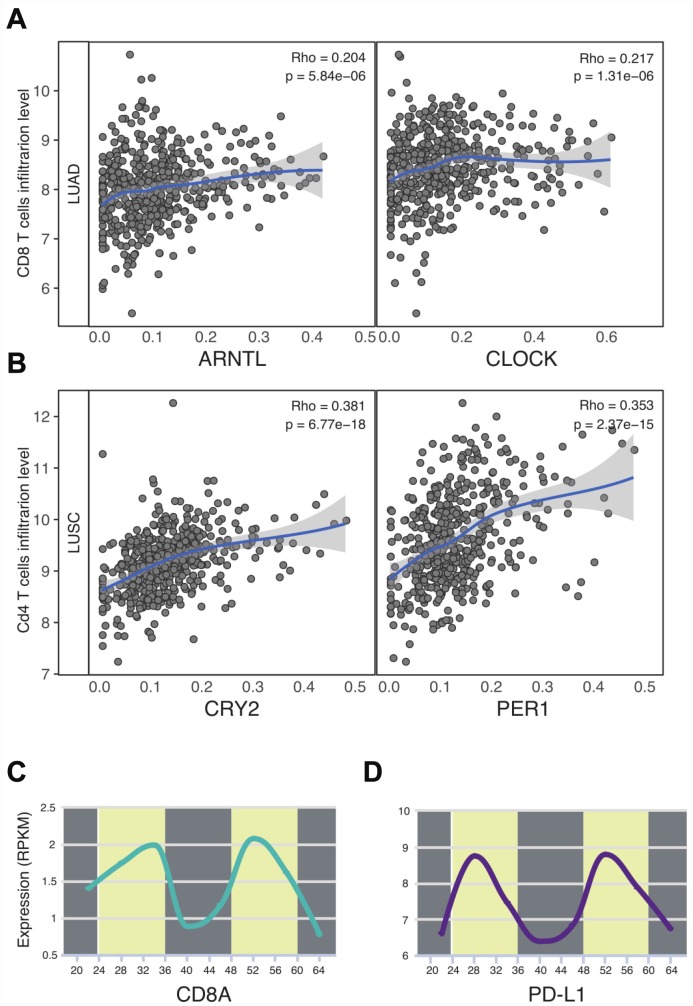
**Circadian clock associates with the immune infiltrates and PD-L1 immune checkpoint.** (**A**, **B**) Circadian genes were correlated with CD8 T cells and CD4 T cells. (**C**) CD8A expression was time-dependent. (**D**) The circadian rhythm of PD-L1. Abbreviations: LUAD, lung adenocarcinoma; LUSC, lung squamous cell carcinoma.

The immune checkpoint inhibitor has profoundly changed the landscape of thoracic cancer treatments. A recent paper shed light on the circadian rhythm of immune checkpoints in sepsis [13]. Yet, in the context of cancer, the impact of circadian clock on immune checkpoints and immunotherapy still remains unclear. In order to characterize the tumor microenvironment features in thoracic cancers, we first computed the infiltration level of CD8 T cells as is described before [20]. Unexpectedly, our results highlight the biological role of circadian clock on immune functions in LUAD and LUSC. Especially in LUAD patients, two essential daytime circadian clock genes were all correlated with the infiltration level of CD8 T cells, which are the crucial target of immune checkpoint inhibitors. As for LUSC patients, CRY2 and PER1 were observed to correlate with CD4 T cells. In order to confirm our results, we computed the circadian rhythm of CD8 T cells (CD8A) in lung tissues (P = 3.73e-03) [16]. We also sought to calculate other immune cell markers, however, we cannot retrieve the available data. Next, we identified the circadian rhythm of immune checkpoint PD-L1 (Figure 4D). Surprisingly, in normal lung tissues, the PD-L1 expression was time-dependent (P = 2.33e-03). These data supports the possible association between the circadian clock and immune infiltrates in thoracic cancers.

## DISCUSSION

In this report, we used multi-omics tools in order to explore the role of circadian clock in thoracic cancers. Especially in lung adenocarcinoma and lung squamous cell carcinoma patients, circadian clock activates the oncogenic signalling pathways, correlates with the prognosis, and affects the immune infiltrates. Strikingly, circadian clock widely impact the CD8 T cells in lung adenocarcinoma and the expression of PD-L1, suggesting that response to immune checkpoint inhibitor might be time-dependant. Our study also reveals that tumor microenvironment in “day-time” and “night-time” may vary.

The expression of core circadian clock molecules could largely define the circadian clock state [23]. Under the circumstance that CLOCK and BMAL1 are lower expressed, the tumor microenvironment is in the state of “nighttime”. Our results showed that a high fraction of clock molecules are epigenetically and transcriptionally down-regulated in LUAD and LUSC samples. In such cases, circadian rhythms were disrupted. Tumors have their specific circadian rhythms. Hence, targeting tumor-specific circadian clock might be a useful strategy of developing anti-cancer agents.

Furthermore, the expression alterations in circadian molecules are associated with the activity of oncogenic pathways. For example, our results highlight the inhibitory effects of core circadian on the cell cycle. The circadian clock and cell cycle are different biological circuits with multiple layers of cooperative regulation. In accordance with previous studies [18], our observation confirmed the crosstalk between circadian rhythm and cell cycle. On the other side, we also find previously-unknown clock-associated signalling pathways. RTK pathway, which are critical for numbers of cellular processes including cancer cell proliferation, migration, and invasion, was proved to be regulated by circadian clock. This finding underlies further exploration on circadian timing of RTK inhibitor [19] for lung cancer treatment.

Our analysis do have some limitations. We did not control the time at which the data are collected from different patients. In fact, several factors can regulate clock genes such as environmental and genetic factors. We could not control these factors simultaneously and our present conclusion may be affected by certain biases. Another concern is that CD8A molecules are expressed on other types of immune cells but not limited to CD8 T cell. In the future, experimental improvements such as the use of the more convenient sample collection system, may enable more detailed time-series analysis. The chrono-immunotherapy strategy may only be regarded as hypothesis-generating and more evidence from experimental data is need to further explore this idea.

Finally, our intriguing observation revealed that most actionable genes tightly associated with core circadian molecules. In other words, circadian timing in those cancer therapies may potentially improve the effectiveness and safety. By using Cancer Therapeutics Response Portal, we profiled the circadian clock-linked drugs and computed a list of timing-actionable drug candidates. Unexpectedly, we not only found the circadian rhythm of certain molecules but also observed the timing-dependent feature of certain immune cells. Applying immune checkpoint inhibitor following circadian timing may potentially improve the efficacy and reduce the severe side effects in the clinical setting.

## MATERIALS AND METHODS

### Data sets and data availability

We include patient samples of lung adenocarcinoma (LUAD), lung squamous cell carcinoma (LUSC), and esophageal carcinoma (ESCA) from The Cancer Genome Atlas (https://cancergenome.nih.gov). We download the clinical information of all patients from UCSC Xena (https://xenabrowser.net/hub/). We collected the Illumina human methylation, single nucleotide variation, and mRNA normalized RPKM values from LUAD, LUSC, and ESCA patients (n = 576, 533, and 196, respectively). The patient would be removed out if the corresponding data are absent. Heat maps were designed based on pheatmap package.

### Immune infiltrate signature construction and pathway activity analysis

The signature of immune cell level (B cells, CD4+ T cells, CD8+ T cells, Neutrophils, Macrophages and Dendritic cells) was computed as is described in a previous study [20]. In order to fetch the pathway activity score (PAS) of each sample, we first download the Reverse phase protein array value from The Cancer Proteome Atlas (http://tcpaportal.org/tcpa/). We focused on those hallmark pathways, including TSC/mTOR, RTK, RAS/MAPK, PI3K/AKT, Hormone ER, Hormone AR, EMT, DNA Damage Response, Cell Cycle, and Apoptosis. RPPA data were normalized by standard deviation, and the score of each equals to the relative protein level of all positive-regulatory molecules minus negative-regulatory molecules [21].

### Gene expression and correlation analysis

All related analysis was based on R. The differential expression analysis was calculated based on |Fold Change| > 1 and FDR ≤ 0.05. The survival analysis was based on Xena and Survminer. The drug sensitivity analysis was based on Cancer Therapeutics Response Portal (https://portals.broadinstitute.org/ctrp/). To analyze the correlation of expression and drug sensitivity, the Pearson correlation coefficients of transcript levels and AUCs was used as was described before [22]. In order to define genes with pathway activities, we first divided gene expression into 2 groups (high and low expression). When the PAS of Gene A high group is larger than the PAS of Gene A low group, we consider gene A may have a activation impact on that pathway [23].

### Copy number analysis and methylation analysis

The CNV was divided into two types including heterozygous CNV and homozygous CNV, which marks the occurrence of CNV on only one or two chromosomes. Only genes with > 5% CNV in cancers will show corresponding point on the figure. The significant differential methylation was defined as FDR < 0.05.

### Survival analysis

Combining gene expression and survival profiles of each sample, we use median RPKM of each mRNA to divide patients into high-risk and low-risk groups. By using survival package, Kaplan-Meier curves were designed and log-rank P values were computed for every single gene.

## Supplementary Material

Supplementary Figures
