# Supplementary material for: Circadian clock associates with tumor microenvironment in thoracic cancers
Source: Aging (Albany NY). 2019 Dec 23;11(24):11814–28. doi: 10.18632/aging.102450 (PMC6949103; doi:10.18632/aging.102450)
Supplement: Supplementary Figures [file aging-11-102450-s001..pdf]

SUPPLEMENTARY FIGURES

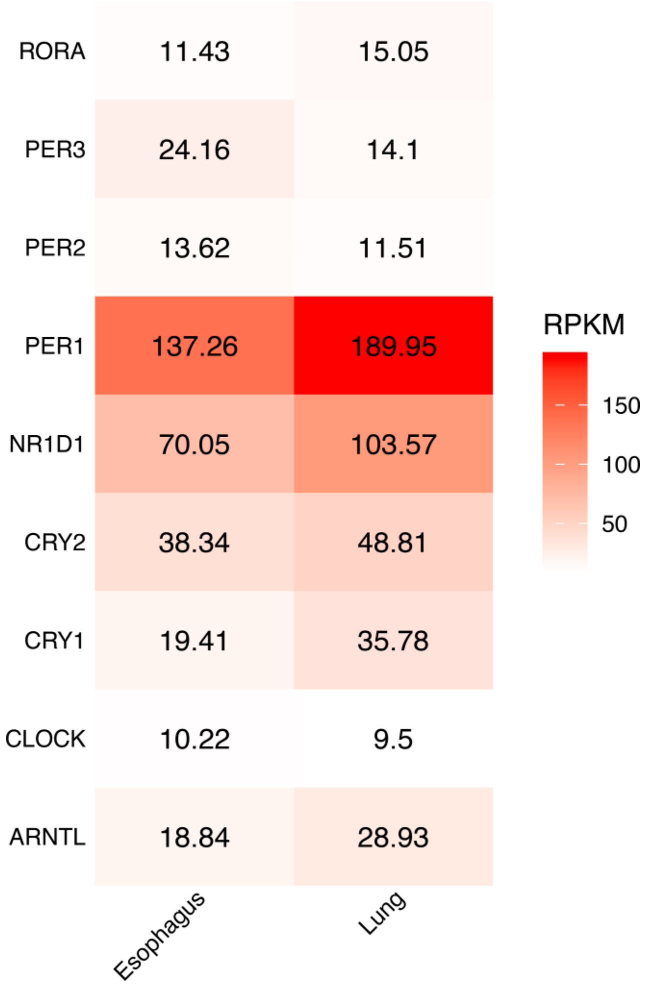

Supplementary Figure 1. Core circadian clock expression in esophagus and lung tissues from GTEx dataset.

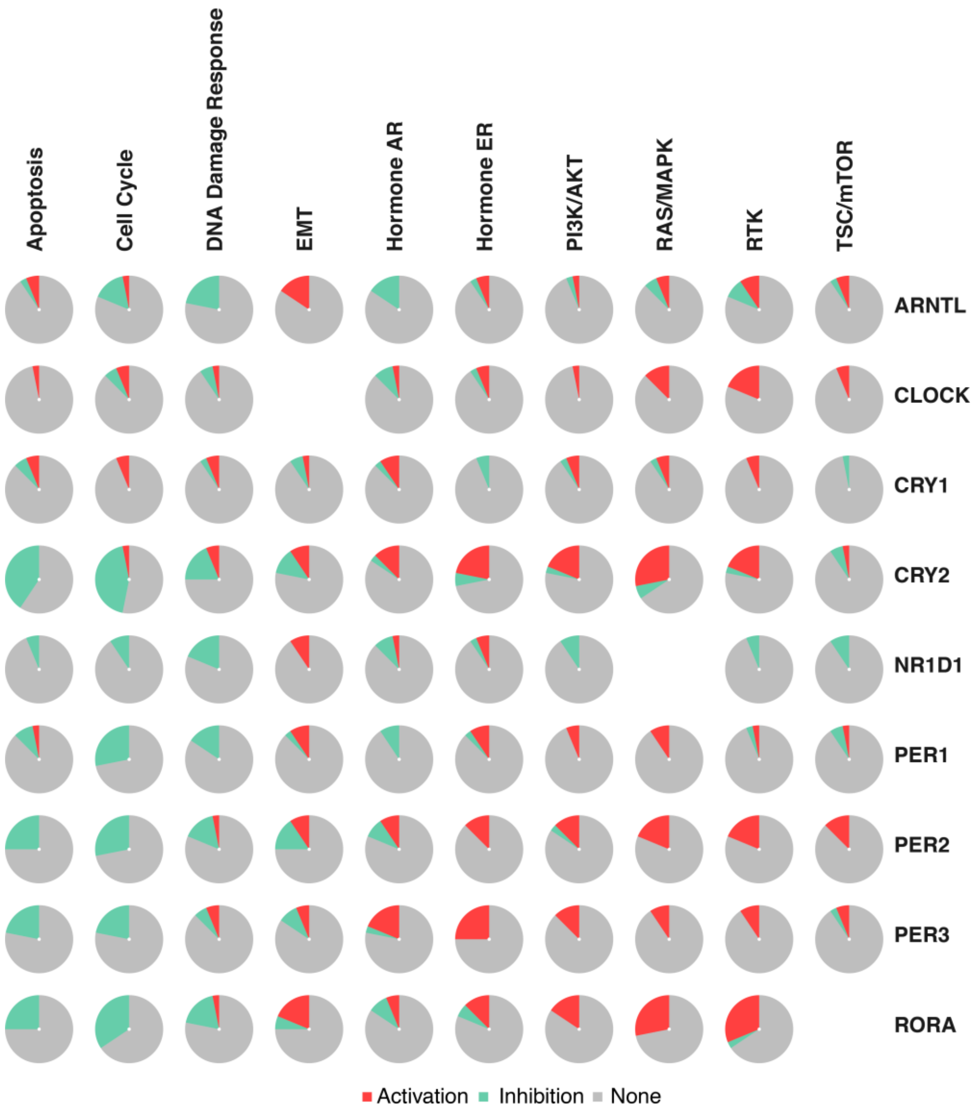

Supplementary Figure 2. Core circadian clock genes correlates with cancer hallmarks in thoracic cancers.

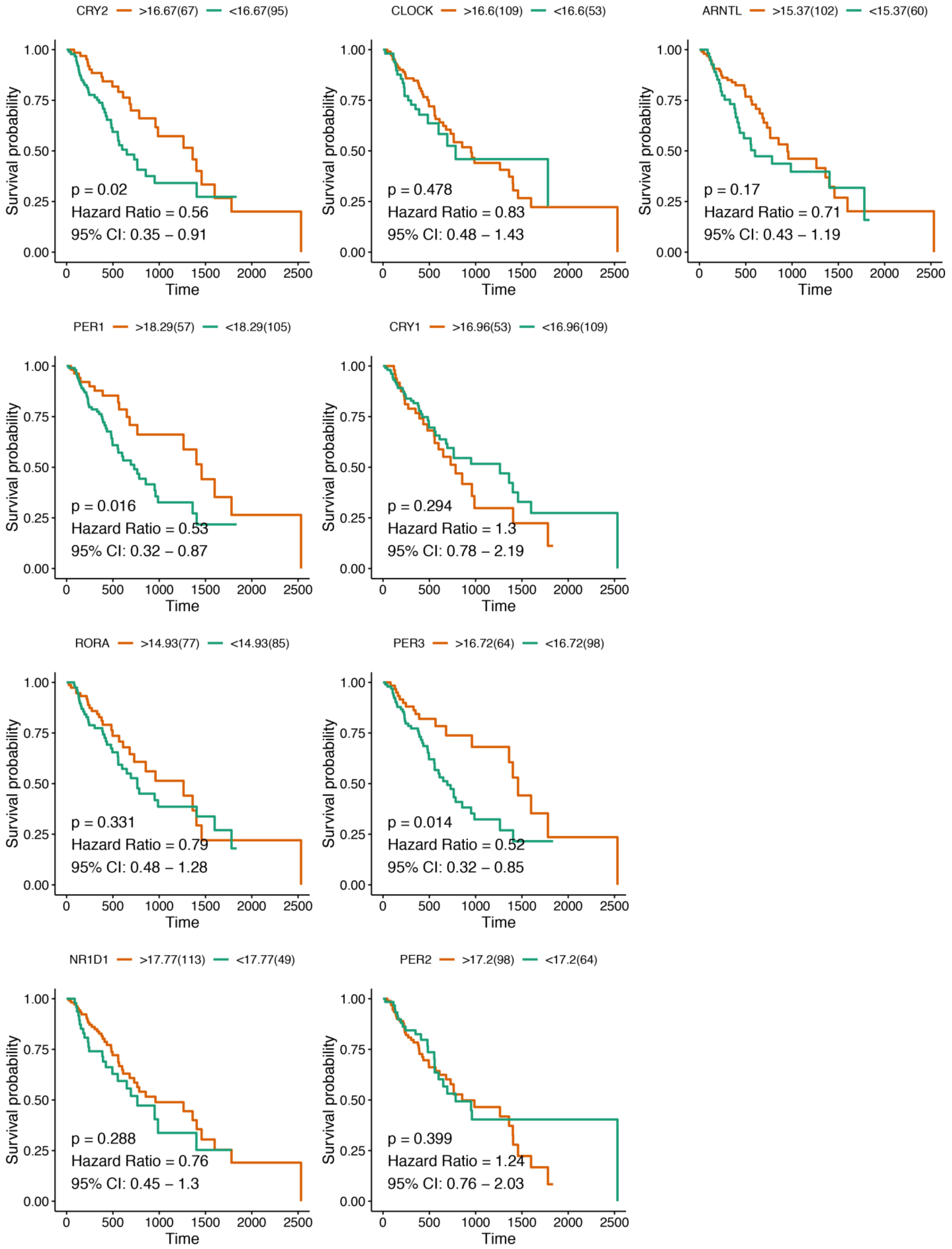
CRY2 >16.67(67) <16.67(95)
p = 0.02
Hazard Ratio = 0.56
95% CI: 0.35 – 0.91
CLOCK >16.6(109) <16.6(53)
p = 0.478
Hazard Ratio = 0.83
95% CI: 0.48 – 1.43
ARNTL >15.37(102) <15.37(60)
p = 0.17
Hazard Ratio = 0.71
95% CI: 0.43 – 1.19
PER1 >18.29(57) <18.29(105)
p = 0.016
Hazard Ratio = 0.53
95% CI: 0.32 – 0.87
CRY1 >16.96(53) <16.96(109)
p = 0.294
Hazard Ratio = 1.3
95% CI: 0.78 – 2.19
RORA >14.93(77) <14.93(85)
p = 0.331
Hazard Ratio = 0.79
95% CI: 0.48 – 1.28
PER3 >16.72(64) <16.72(98)
p = 0.014
Hazard Ratio = 0.52
95% CI: 0.32 – 0.85
NR1D1 >17.77(113) <17.77(49)
p = 0.288
Hazard Ratio = 0.76
95% CI: 0.45 – 1.3
PER2 >17.2(98) <17.2(64)
p = 0.399
Hazard Ratio = 1.24
95% CI: 0.76 – 2.03
Survival probability
1.00
0.75
0.50
0.25
0.00
0
500
1000
1500
2000
2500
Time

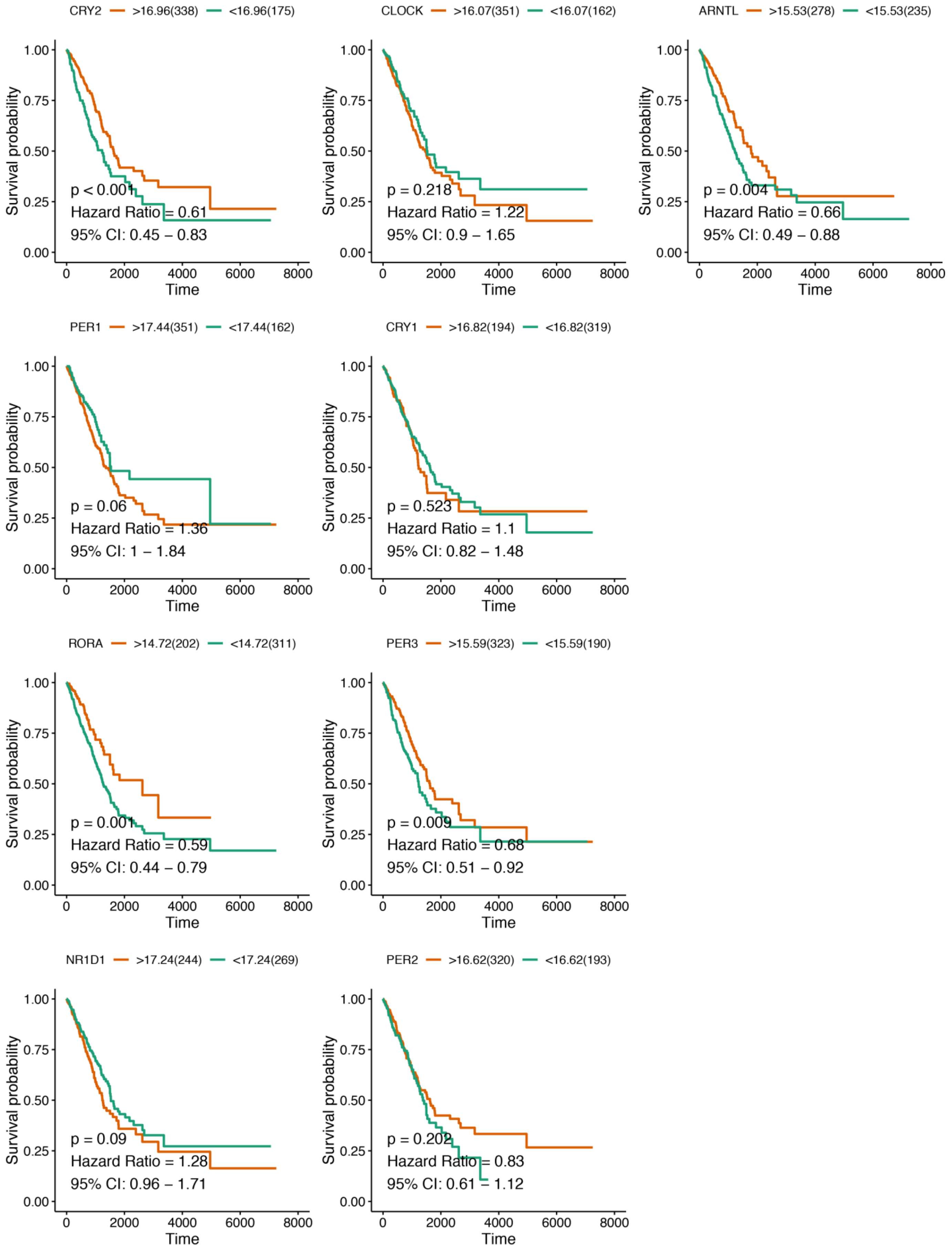
CRY2 >16.96(338) <16.96(175)
p < 0.001
Hazard Ratio = 0.61
95% CI: 0.45 – 0.83
CLOCK >16.07(351) <16.07(162)
p = 0.218
Hazard Ratio = 1.22
95% CI: 0.9 – 1.65
ARNTL >15.53(278) <15.53(235)
p = 0.004
Hazard Ratio = 0.66
95% CI: 0.49 – 0.88
PER1 >17.44(351) <17.44(162)
p = 0.06
Hazard Ratio = 1.36
95% CI: 1 – 1.84
CRY1 >16.82(194) <16.82(319)
p = 0.523
Hazard Ratio = 1.1
95% CI: 0.82 – 1.48
RORA >14.72(202) <14.72(311)
p = 0.001
Hazard Ratio = 0.59
95% CI: 0.44 – 0.79
PER3 >15.59(323) <15.59(190)
p = 0.009
Hazard Ratio = 0.68
95% CI: 0.51 – 0.92
NR1D1 >17.24(244) <17.24(269)
p = 0.09
Hazard Ratio = 1.28
95% CI: 0.96 – 1.71
PER2 >16.62(320) <16.62(193)
p = 0.202
Hazard Ratio = 0.83
95% CI: 0.61 – 1.12
Survival probability
Time

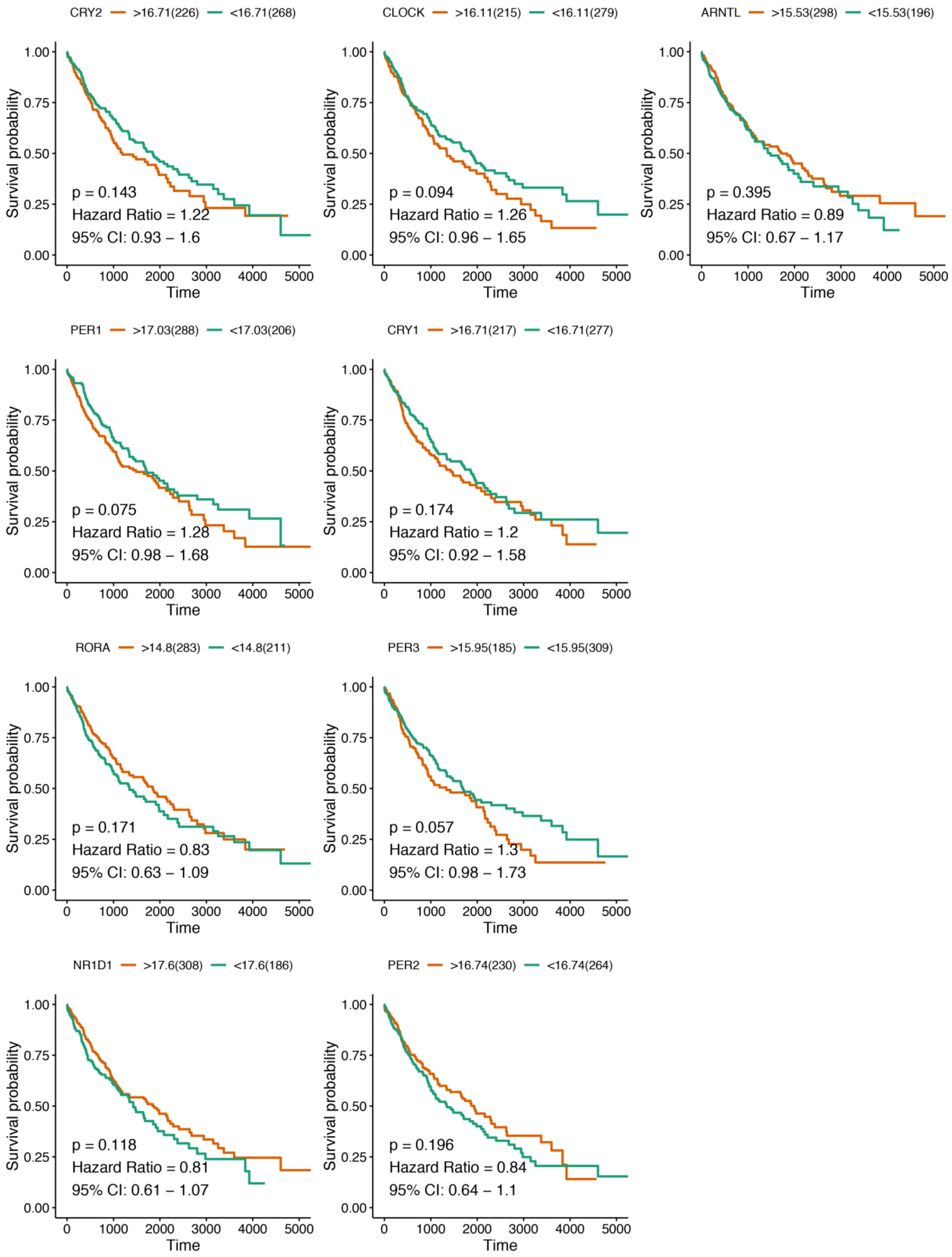

Supplementary Figure 3. Circadian clock impacts the prognosis in ESCA, LUAD, and LUSC patients. log-rank test was used.

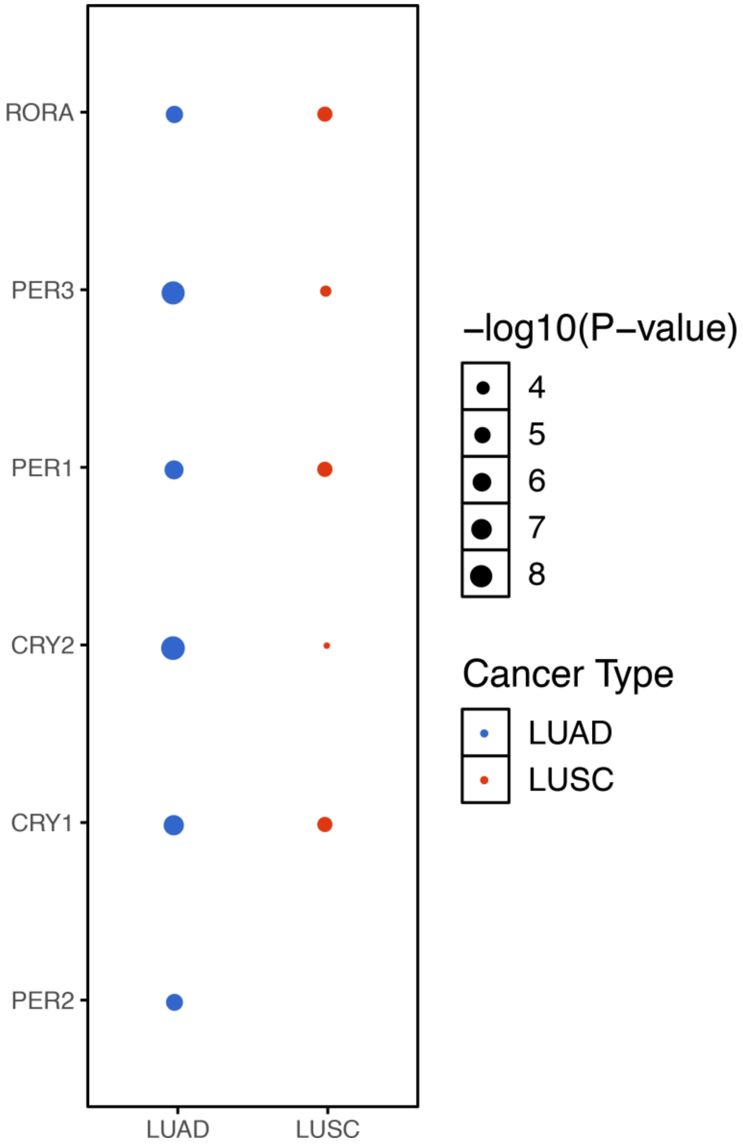

Supplementary Figure 4. Core circadian clock gene expression varies in LUAD and LUSC subtypes.
